# AOJNMB is Indexed in PubMed Central from the First Issue

**DOI:** 10.22038/aojnmb.2016.8030

**Published:** 2017

**Authors:** S. Rasoul Zakavi

**Affiliations:** Editor in Chief, AOJNMB

AOJNMB has a very successful year in 2016 and is indexed in PubMed Central from the first issue. Now AOJNMB could easily be searched in NLM database and full text of articles could freely be downloaded in HTLM or PDF format from PubMed Central. I am deeply grateful to many scientists and physicians who contributed to this success either by publishing their excellent works in AOJNMB or providing critical scientific review of the submitted manuscripts, introduction of the AOJNMB in their countries and sending me their suggestions and comments for improvement of the journal. My special thanks go to our Japanese colleagues who submitted the highest number of manuscripts to AOJNMB in the last few years. Currently we have submissions from more than 20 different countries and I am looking forward to publishing more research works from other countries in the region especially from those who have a solid research system in place. Being indexed in PubMed Central, dramatically improved the visibility of the journal and it is reflected in the increased number of submissions in the last few months. The number of submission to AOJNMB has nearly doubled in the 2^nd^ half of 2016 and submissions from European colleagues have significantly increased too. AOJNMB is also included in databank of Geneva Foundation for Medical Education and Research (GFMER) and will continue application for inclusion in all indexing databanks.

Improvement in visibility of the journal may result in increasing number of citation. AOJNMB had 69 citations in Google scholar for 77 citable published articles that is very good for a newly published journal. It is a great honor that AOJNMB has been cited in more than 30 different journals including the most important journals in the field of Nuclear Medicine like; The Journal of Nuclear Medicine, European Journal of Nuclear Medicine and Molecular Imaging Physics, Seminars in Nuclear Medicine, Annuals of Nuclear Medicine, Nuclear Medicine and Biology, and Clinical Nuclear Medicine (1-6). One of the articles of AOJNMB got more than 10 citations and the H index of the journal has increased to 4.

The future is bright for AOJNMB and we are looking forward to being indexed in ISI web of knowledge. We will do our best to serve as a reliable and valid publication forum for physicians and scientists in the field of nuclear medicine and molecular imaging.

It is an immense pleasure for me to take this opportunity for Season’s greetings and wish all of our readers a Merry Christmas and a Happy New Year 2017.
